# Repositioning of glaucoma tubes into the pars plana for refractory malignant glaucoma: a case report

**DOI:** 10.1186/1752-1947-7-102

**Published:** 2013-04-11

**Authors:** Julia Song, Alessandro Castellarin, Michael Song, Alice Song

**Affiliations:** 1 Long Beach Memorial Medical Center, 2840 Long Beach Blvd. , #330, Long Beach, CA 90806, USA; 2 California Retina Consultants and Research Foundation, 515 East Micheltorena, Suite C, Santa Barbara, CA 93103, USA

**Keywords:** Ciliary block glaucoma, Malignant glaucoma, Pars plana, Pars plana vitrectomy, Tube

## Abstract

**Introduction:**

Malignant glaucoma occurs when the intraocular pressure elevates in the setting of a shallow anterior chamber and patent iridectomy. We describe a case in which malignant glaucoma that was refractory to conventional treatment and complete vitrectomy was successfully managed by rerouting the glaucoma tubes into the pars plana.

**Case presentation:**

A 47-year-old Latino man had a history of neovascular glaucoma and subsequent peripheral anterior synechiae. He was status post-two glaucoma drainage devices. He developed pupillary block. Laser iridotomy was performed without complications. He subsequently developed malignant glaucoma that was refractory to yttrium aluminum garnet capsulohyaloidotomy of the anterior hyaloid. He underwent pars plana vitrectomy with successful control of his intraocular pressure. After 2 weeks, the malignant glaucoma recurred. He underwent repositioning of the tubes into the pars plana with successful control of his intraocular pressure.

**Conclusion:**

In rare cases of malignant glaucoma refractive to yttrium aluminum garnet hyaloidotomy and vitrectomy, placement of glaucoma drainage devices is a reasonable alternative.

## Introduction

Malignant glaucoma was first introduced in 1869 by Dr von Graefe. It is a condition consisting of elevated intraocular pressure (IOP) with shallow anterior chamber despite a patent iridectomy. Risk factors include hyperopia, narrow angle, and a history of miotic use. It can occur following any intraocular surgery [1] (cataract or glaucoma) or laser procedures [2,3] and has also been reported in eyes that have not undergone any procedures. It is typically treated medically with cycloplegia, aqueous suppression, and osmotic agents, or with neodymium-doped yttrium aluminum garnet (YAG) laser capsulo-hyaloidotomy or surgical disruption of the anterior hyaloid [4]. In cases that are refractory to the above, pars plana vitrectomy with or without lensectomy has been used [3,5-10].

We report a case in which a patient who was refractory to pars plana vitrectomy underwent tube repositioning into the pars plana cavity. There is one other case report of a single tube insertion into the pars plana with vitrectomy [11]. This is the first reported case of rerouting two tubes from the anterior chamber into the pars plana for the treatment of malignant glaucoma after the patient’s malignant glaucoma recurred following pars plana vitrectomy.

## Case presentation

A 47-year-old Latino man with poorly controlled diabetes developed neovascular glaucoma in both eyes (OU) with maximal IOPs in the 40mmHg range OU. His past ocular history was significant for proliferative diabetic retinopathy, s/p panretinal photocoagulation lasers OU and multiple Avastin® (bevacizumab) injections by the retina specialist. The patient was on maximal medications. He had undergone glaucoma drainage devices OU with Baerveldt® 101–350 (Abbott Medical Optics, Inc.) to his right eye (OD) and left eye (OS) 5 months later. His IOP OD elevated again despite an addition of five glaucoma medications and oral acetazolamide, so a second glaucoma drainage device was placed OD in the inferotemporal quadrant.

One year later, his IOPs were 8mmHg OD and 16mmHg OS. His visual acuity OD was 20/200. He was taking maximal medications OU again. His visual acuity OD worsened to count fingers vision OD. He underwent cataract extraction OD, which improved his visual acuity to 20/125. His IOPs were maintained in the mid-teens range; he refused further glaucoma surgery until his visual fields worsened to 3° OD and 6° OS. Oral acetazolamide 500mg twice a day was added. He continued to refuse surgical intervention despite worsening visual fields.

**Figure 1:**
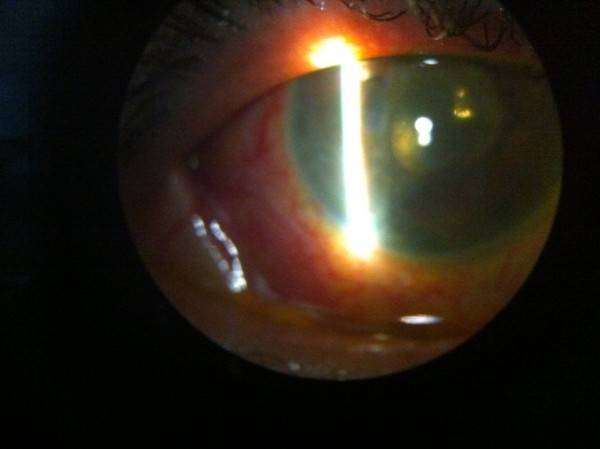
Slit lamp photo of patient following placement of glaucoma tubes into the pars plana.

Six months later, he developed iris bombe OD. He underwent laser iridotomy, which lowered his IOP to 12mmHg. Two weeks later, his IOP OD increased to 80mmHg. He had a flat anterior chamber, with iris enveloping both the superotemporal and inferotemporal glaucoma tubes. Neither cycloplegia nor YAG capsulo-hyaloidotomy OD helped to lower his IOP or deepen his anterior chamber. He underwent emergent pars plana vitrectomy OD with removal of the anterior hyaloid that evening. His anterior chamber deepened significantly, and his IOPs lowered to 12mmHg. He was placed on topical steroids every 2 hours and was stable for 2 weeks with a patent iridotomy. His IOP rose again to 80mmHg with a flat anterior chamber despite a patent iridotomy. He underwent rerouting of both glaucoma tubes into the pars plana cavity that evening. The next day, his IOP was 11mmHg with a deep anterior chamber (Figure 1). His IOP has remained stable since then with the addition of glaucoma medications and cycloplegic agents; however, his visual acuity had decreased to hand motions.

## Discussion

Pars plana vitrectomy has been found to be efficacious in the treatment of malignant glaucoma following cataract extraction [9,10]. Pars plana vitrectomies have either been combined with cataract extraction and posterior capsulectomy [12] or combined with trabeculectomy [13] in select patients who have significant peripheral anterior synechiae. Cekic and Batman [14] recommended that concomitant lensectomy be performed in order to save the patient a second surgical procedure in the future. Tube placement into the pars plana at the same time as pars plana vitrectomy has been reported previously [11].

This is the second case of a tube implant in the pars plana and the first reported incidence of repositioning glaucoma tubes into the pars plana cavity to treat malignant glaucoma. There has been a reported case [15] of a patient's eye having recurrent malignant glaucoma despite having undergone vitrectomy in which the anterior hyaloid was left intact. Our patient’s iridotomy was patent despite significant posterior synechiae. It is possible that he developed an inflammatory membrane over the iridotomy; however, the iridotomy was patent. Our patient had had a complete vitrectomy and removal of the anterior hyaloid. In addition the original iridotomy was enlarged using the vitreous cutter probe via pars plana. However, rerouting of the tubes into the posterior cavity, in close proximity to where aqueous production occurs, helped break the cycle of malignant glaucoma.

## Conclusions

This report demonstrates that patients who are refractory to conventional treatment for malignant glaucoma can benefit from rerouting of the glaucoma tube(s) into the pars plana; this can be done at the same time as the vitrectomy if the risk for further visual deterioration is high.

## Consent

Written informed consent was obtained from the patient for publication of this case report and any accompanying images. A copy of the written consent is available for review by the Editor-in-Chief of this journal.

## Competing interests

We, the authors, JS, MS, and AS declare that we have no competing interests. One of the authors, AC, has competing interests in the following: Genentech (lecturer and consultant), Alcon (stocks), Alimera Science (consultant) and QLT/Novartis (consultant).

## Authors’ contributions

JS and AC examined the patient before and after treatment. MS and AS performed the literature search. All authors read and approved the final manuscript.
